# Hepatic SATB1 induces paracrine activation of hepatic stellate cells and is upregulated by HBx

**DOI:** 10.1038/srep37717

**Published:** 2016-11-24

**Authors:** Jin Gong, Wei Tu, Jian Han, Jiayi He, Jingmei Liu, Ping Han, Yunwu Wang, Mengke Li, Mei Liu, Jiazhi Liao, Dean Tian

**Affiliations:** 1 Department of Gastroenterology, Tongji Hospital of Tongji Medical College, Huazhong University of Science and Technology, Wuhan, 430030, China; 2 Department of Pediatrics, Tongji Hospital of Tongji Medical College, Huazhong University of Science and Technology, Wuhan, 430030, China

## Abstract

Chronic hepatitis B virus (HBV) infection is a major cause of chronic liver diseases, but its involvement in hepatic fibrogenesis remains unclear. Special AT-rich binding protein 1 (SATB1) has been implicated in reprogramming chromatin organization and transcription profiles in many cancers and non-cancer-related conditions. We found that hepatic SATB1 expression was significantly up-regulated in fibrotic tissues from chronic hepatitis B virus (HBV)-infected patients and HBV transgenic (HBV-Tg) mouse model. Knockdown of SATB1 in the liver significantly alleviated CCl4-induced fibrosis in HBV-Tg mouse model. Moreover, we suggested HBV encoded x protein (HBx) induced SATB1 expression through activation of JNK and ERK pathways. Enforced expression of SATB1 in hepatocytes promoted the activation and proliferation of hepatic stellate cells (HSCs) by secretion of connective tissue growth factor (CTGF), Interleukin-6 (IL-6) and platelet derived growth factor-A (PDGF-AA). Our findings demonstrated that HBx upregulated hepatic SATB1 which exerted pro-fibrotic effects by paracrine activation of stellate cells in HBV-related fibrosis.

Chronic liver injury is one of the major public health problems worldwide, mostly resulting in progressive hepatic fibrosis, which is characterized by excessive production and deposition of extracellular matrix (ECM) in the liver. It is well accepted that the activation of resident hepatic stellate cells (HSCs) into fibroblast-like cells is a hallmark of hepatic fibrogenesis^1^. Acitvated HSCs trigger the expression of α-smooth muscle actin (α-SMA) and production of abnormal ECM, along with enhanced proliferation and migration^2^. However, recent developments challenge the role of HSC and highlight that hepatocyte acts as an active participant in liver fibrogenesis. Several studies show that the impaired hepatocytes could contribute to progressive fibrosis by remodeling ECM and interacting with surrounding cells, particularly HSCs and intrahepatic immune cells^3^,^4^,^5^,^6^. Apoptotic hepatocytes are reported to release some endogenous compounds such as damage associated molecular patterns (DAMPs) and apoptotic bodies, leading to HSC activation within the liver^7^,^8^.

Chronic hepatitis B virus (HBV) infection is a major cause of hepatic fibrosis^9^,^10^. There is convincing evidence showing that HBV encoded x antigen (HBxAg) strongly correlates with the severity of chronic liver diseases (CLD) and the development of fibrosis. Overexpression of HBx induces lipid accumulation in HBx-transgenic mice^11^. HBxAg could alter the production of the extracellular matrix by modulating the expression of several matrix metalloproteins (MMPs) and fibronectin^12^,^13^. Besides, HBx mediates activation of HSCs by paracrine production of pro-fibrotic factor TGFB1 and recruitment of Th17 cells^14^,^15^,^16^,^17^,^18^; nevertheless, the role of HBV-infected hepatocytes in hepatic fibrogenesis remains elusive.

Special AT-rich binding protein 1 (SATB1), a nuclear matrix attachment regions (MARs)-binding protein, is found predominantly in thymocytes. SATB1 regulates gene expression by recruiting chromatin remodeling complexes and tethering specialized DNA sequences^19^,^20^. Previous studies revealed SATB1 was critical for the development and maturation of thymocytes and T cells^21^. SATB1 is also involved in rapid induction of multiple cytokines genes on T-helper 2 cell activation^22^. Recent reports show that SATB1 is correlated with metastatic phenotypes and poor clinical prognosis in various tumors^23^,^24^,^25^,^26^. Consistent with other reports, our former study revealed that SATB1 promoted development and progression of liver cancer by regulation of genes related to cell cycle, apoptosis and EMT^27^,^28^. We also observed the protective effect of SATB1 in hepatic fibrogenesis by regulation of HSC activation^29^. However, whether SATB1 in impaired hepatocytes exerts an effect on liver fibrosis remains unknown.

In this study, we clarify the role of SATB1 in HBV-related hepatic fibrogenesis and elucidate a cross talk between hepatocytes and HSCs through secretion of profibrogenic cytokines IL-6, PDGF-AA and CTGF induced by hepatic SATB1.

## Results

### SATB1 is upregulated in hepatitis B-related liver fibrosis

**Figure 1:**
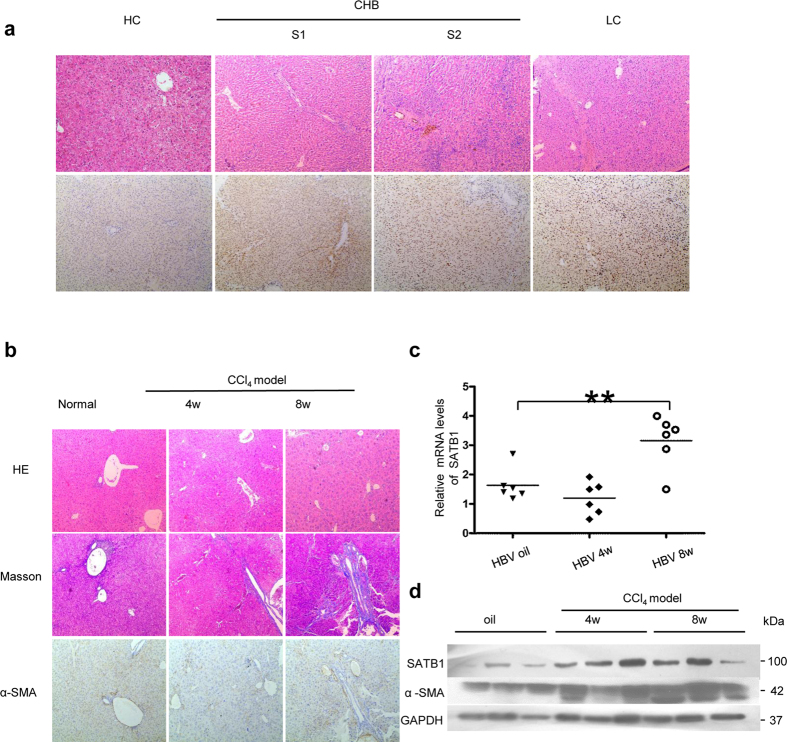
SATB1 is upregulated in hepatitis B-related liver fibrosis. (**a**) Representative immunohistochemical (IHC) staining of SATB1 and H&E staining in liver from a healthy control (HC) subject and chronic hepatitis B (CHB) and liver cirrhosis (LC) patients. (100×). (**b**) IHC was used to determine the expression of α-SMA in CCl4-induced fibrosis in HBV-Tg mouse model. H&E and Masson’s trichrome staining showed pathological conditions and collagen deposition. (100×). (**c**) Real-time PCR showed the expression of SATB1 in HBV-Tg mouse model induced by CCl4 for 4 and 8 weeks (n = 6 in each group). **P < 0.01 vs. normal control group by Student’s t-test. (**d**) Western blot analyses of SATB1 and α-SMA in the fibrotic livers from HBV-Tg mouse model (n = 3 in each group). GAPDH was used as a loading control. Full-length blots are included in the Supplementary Fig. S5.

Immunohistochemical (IHC) staining was used to investigate SATB1 expression in liver tissues from chronic hepatitis B (CHB) and liver cirrhosis (LC) in HBV-infected patients. Our results showed that endogenous SATB1 was rarely detected in normal liver tissues, while positive staining of SATB1 was mainly observed in the nucleus of hepatocytes from HBV-infected samples (Fig. 1a). Further analysis of 68 cases of patients IHC staining for SATB1 expression showed that SATB1 was significantly upregulated in CHB and LC patients (Table 1). Spontaneous liver fibrosis was previously reported in HBV transgenic (HBV-Tg) mice C57BL/6J-TgN(AlblHBV) 44Bri, and HBV-Tg mice were shown oversensitive to CCl4-induced liver injury^18^,^30^. Therefore, we established a CCl4-induced fibrosis model in HBV transgenic mice, confirmed by hematoxilin/eosin (H&E) staining, Masson’s trichrome staining and α-SMA expression (Fig. 1b). Similarly, the mRNA and protein levels of SATB1 were both enhanced in CCl4-induced fibrosis model, compared with the control group (Fig. 1c,d), yet mRNA level of SATB1 in CCl4-induced fibrosis model at 4 weeks was not significantly increased (Fig. 1c).

### SATB1 silencing attenuates hepatic fibrosis in HBV transgenic mice

**Figure 2:**
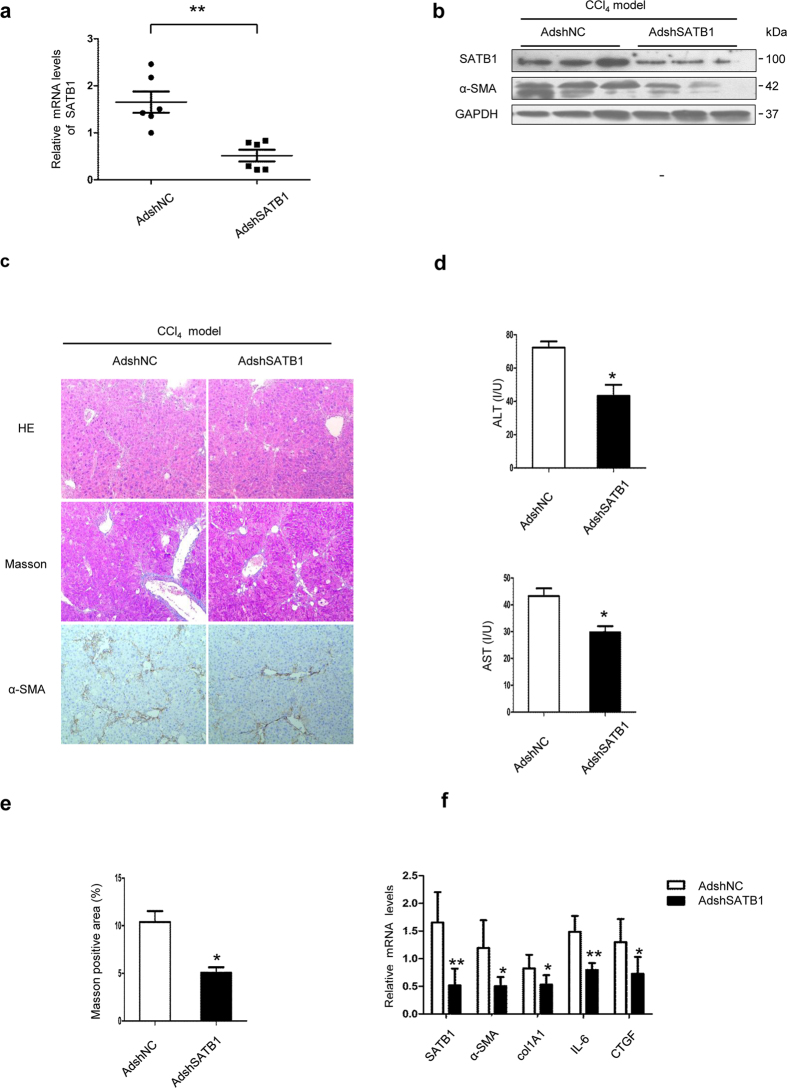
Knockdown of SATB1 ameliorates CCl4-induced fibrosis in HBV-Tg mouse model. (**a**) Adenovirus carrying shRNA against SATB1 (AdshSATB1) or negative control (AdshNC) was injected into HBV-Tg mouse model. Inhibition efficiency of SATB1 expression was examined by real-time PCR in the fibrotic livers from AdshSATB1 or AdshNC-treated mice (n = 6 in each group). **P < 0.01 vs. AdshNC group. (**b**) Western blot was used to detect the protein levels of SATB1 and α-SMA in liver tissues (n = 3 in each group). Full-length blots are included in the Supplementary Fig. S6. (**c**) Representative IHC staining was used to determine the expression of α-SMA in the fibrotic liver. H&E and Masson’s trichrome staining were used to show pathological conditions and collagen deposition (100×). (**d**) Plasmid levels of ALT and AST were detected by elisa Kit (n = 6). *P < 0.05 vs. AdshNC group. (**e**) Semi-quantitative analysis of Masson’s trichrome staining in the fibrotic livers from AdshSATB1 or AdshNC-treated mice (n = 6). *P < 0.05 vs. AdshNC group. (**f**) Real-time PCR was used to detect the expression of SATB1, α-SMA, COL1A1, IL-6, CTGF in liver tissues from HBV-Tg mice. (n = 6). *P < 0.05, **P < 0.01 vs. AdshNC group.

To confirm the role of SATB1 in HBV-related liver fibrosis, we silenced SATB1 *in vivo* in a CCl4-induced fibrosis model of HBV-Tg mice. Adenovirus carrying a SATB1-specific (AdshSATB1) or control shRNA (AdshNC) was given through tail vein injection once a week after CCl4 treatment. Mice were sacrificed 2 weeks after adenovirus administration. Inhibition efficiency of SATB1 was confirmed by real-time PCR and western blot in whole liver extracts (Fig. 2a,b). Our results showed that knockdown of SATB1 led to reduced histological liver damage, as seen by H&E staining (Fig. 2c) and decreased ALT, AST serum levels (Fig. 2d). Furthermore, fibrosis development was attenuated after AdshSATB1 administration, as shown by Masson’s trichrome staining and decreased expression of the fibrotic marker a-SMA (Fig. 2c). Masson staining indicated that liver treated with AdshSATB1 had less ECM deposition (5.1% ± 0.013) compared with AdshNC controls (10.4% ± 0.025) as indicated by reduced ECM area by 50% in the CCl4-induced fibrosis model (Fig. 2e). Besides, the expression of fibrotic markers, including a-SMA, COL1A1 and CTGF as well as proinflammatory cytokines IL-6 were also decreased in AdshSATB1-treated group (Fig. 2f). These data collectively confirm that down-regulation of SATB1 inhibits hepatic fibrogenesis in HBV transgenic mice.

### HBx upregulates SATB1 in hepatic cell line L02

**Figure 3:**
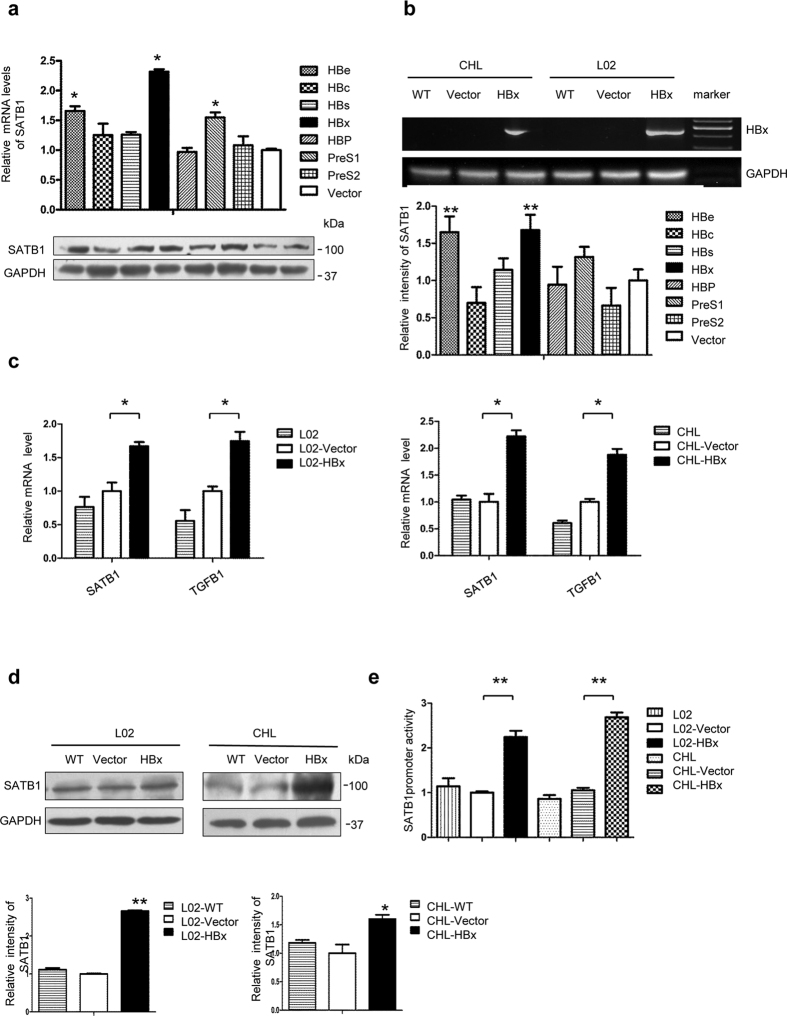
HBx induces SATB1 expression in hepatic cell line L02. (**a**) Real-time PCR and western blot were used to examine SATB1 expression from L02 cells respectively transfected with synthetic DNA plasmids containing seven viral genes of HBV. *P < 0.05, **P < 0.01 vs. Vector group. Full-length blots are included in the Supplementary Fig. S7. (**b**) Identification of HBx from stable HBx-transfected L02 (L02-HBx) and Chang liver cells (CHL-HBx) and corresponding vector control (L02-Vector, CHL-Vector), widetype control (WT) by agarose gelelectrophoresis. Full-length gels are included in the Supplementary Fig. S7. (**c**) The mRNA levels of SATB1 and TGFB1 were analyzed by real-time PCR. (n = 3). *P < 0.05 vs. Vector. (**d**) The expression of SATB1 was analyzed by western blot. Full-length blots are included in the Supplementary Fig. S7. *P < 0.05, **P < 0.01 vs. Vector. (**e**) HBx trans-activated SATB1 promoter activity. SATB1 promoter plasmids were transfected into L02-HBx, CHL-HBx and corresponding control groups. (n = 3). **P < 0.01 vs. Vector.

Next, we transfected synthetic DNA plasmids containing seven viral genes of HBV into human hepatic cell line L02 to screen which viral proteins could effectively upregulate SATB1 expression. It showed that HBx protein markedly increased SATB1 expression, whereas the other viral proteins merely had no effect on SATB1 expression (Fig. 3a). To verify the role of HBx in the modulation of SATB1, we established two stable HBx-transfected cell lines L02 and Chang liver cells (CHL). Chang liver cells were indicated as non-hepatic control (Fig. 3b and Supplementary Fig. S1). Our data showed that expressing of HBx significantly enhanced SATB1 expression at mRNA and protein levels (Fig. 3c,d). To further confirm whether SATB1 gene transcription was induced by HBx, we detected SATB1 promoter activity. As expected, SATB1 promoter activity was increased in HBx-transfected cells compared with controls in both L02 and Chang liver cells (Fig. 3e). Taken together, these results suggest that HBx stimulates the synthesis of SATB1 in liver cells.

### HBx transactivates SATB1 expression through the ERK and JNK pathway

**Figure 4:**
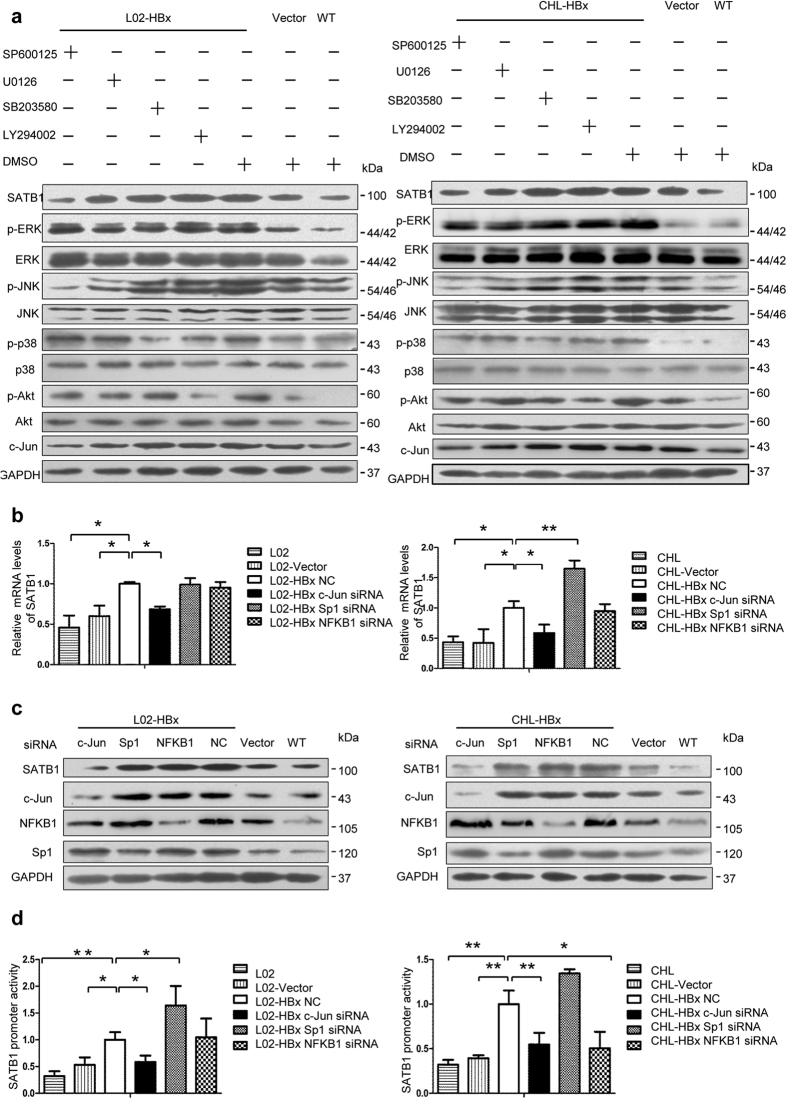
HBx upregulates SATB1 expression through the JNK and ERK pathway and the activation of c-Jun. (**a**) L02-HBx, CHL-HBx cells and corresponding control groups L02-Vector, L02-WT, CHL-Vector, CHL-WT were respectively treated with specific JNK inhibitor SP600125 (25 uM), ERK1/2 inhibitor U0126 (25 uM), PI3K inhibitor Ly294002 (25 uM), p38 inhibitor SB203580 (25 uM) for 48 hours. Protein levels of SATB1, c-Jun, phosphorylated and total JNK, ERK1/2, p38 and Akt were analyzed by western blot. Full-length blots are included in the Supplementary Fig. S8. (**b**) Real-time PCR analysis of SATB1 expression in L02-HBx, CHL-HBx cells and corresponding control groups after transfection with siRNA targeting various transcription factors c-Jun, Sp1, NFKB1 for 48 hours, respectively. (n = 3). *P < 0.05, **P < 0.01 vs. L02-HBx NC or CHL-HBx NC. (**c**) Protein levels of SATB1, c-Jun, Sp1, NFKB1 were analyzed by western blot. Full-length blots are included in the Supplementary Fig. S8. (**d**) SATB1 promoter reporter vectors were contransfected with siRNA targeting various transcription factors c-Jun, Sp1, NFKB1 into L02-HBx and CHL-HBx cells, respectively. The SATB1 promoter activity was measured at 72 h posttranscfection. (n = 3). *P < 0.05, **P < 0.01 vs. L02-HBx NC or CHL-HBx NC.

It is known that HBx can induce the phosphorylation of ERK1/2, JNK and p38 MAPKs^14^. To elucidate the molecule mechanism of HBx-mediated SATB1 expression, U0126, SP600125, SB203580 and LY294002 were used to specifically render inactivate of ERK1/2, JNK,p38 MAPK and PI3k-Akt pathway, respectively. Pretreatment with the ERK1/2 inhibitor (U0126) and JNK inhibitor (SP600125) significantly suppressed HBx-induced SATB1 expression, while pretreatment with p38 or Akt inhibitors had no effect on the SATB1 expression (Fig. 4a and Supplementary Fig. S4a), indicating that ERK1/2 and JNK pathways contributed to HBx-induced SATB1 production. Besides, prominent changes in the levels of c-Jun were noted in HBx-induced SATB1 expression, which suggested that HBx promoted the expression of SATB1 probably in a c-Jun-dependent manner (Fig. 4a). Collectively, our results suggest that HBx may upregulate SATB1 expression via ERK1/2/c-Jun and JNK/c-Jun signal pathways in L02 cell line.

### HBx activates SATB1 promoter in an AP-1-Dependent Manner

It has been reported that HBx could modulate transcription by interacting with various transcription factors, such as activator protein 1 (AP-1), nuclear factor-ĸB (NFKB1), Sp1, ATF/CREB^31^,^32^,^33^,^34^. To examine the mechanism of transcription activation of HBx on the SATB1 promoter, we performed a search of possible transcription-factor-binding sites in the promoter region of SATB1 using JASPAR (http://jaspar.genereg.net/) and TFRESEARCH software (Japanese, ver 1.3). The promoter region of SATB1 contains various binding sites, such as AP-1, NFKB1 and Sp1. Next, we screened a panel of transcription factors including AP-1, NFKB1, Sp1 by delivery siRNA targeting them into HBx-overexpressed cells. It showed that silencing of c-Jun, the subunit of AP-1, obviously abolished enhancement of SATB1 production induced by HBx (Fig. 4b,c and Supplementary Figs S2 and S4b). Importantly, we cotransfected plasmid expressing SATB1 promoter reporter and siRNA targeting transcription factors, c-Jun, NFKB1, Sp1 in stable HBx-transfected L02 and Chang liver cells. Luciferase assay showed that augmenting SATB1 promoter activity mediated by HBx was aboslished when c-Jun was suppressed (Fig. 4d). These data suggests that HBx could upregulate the activity of SATB1 promoter via AP-1.

### SATB1 expression in hepatocytes induces paracrine activation and proliferation of HSCs

**Figure 5:**
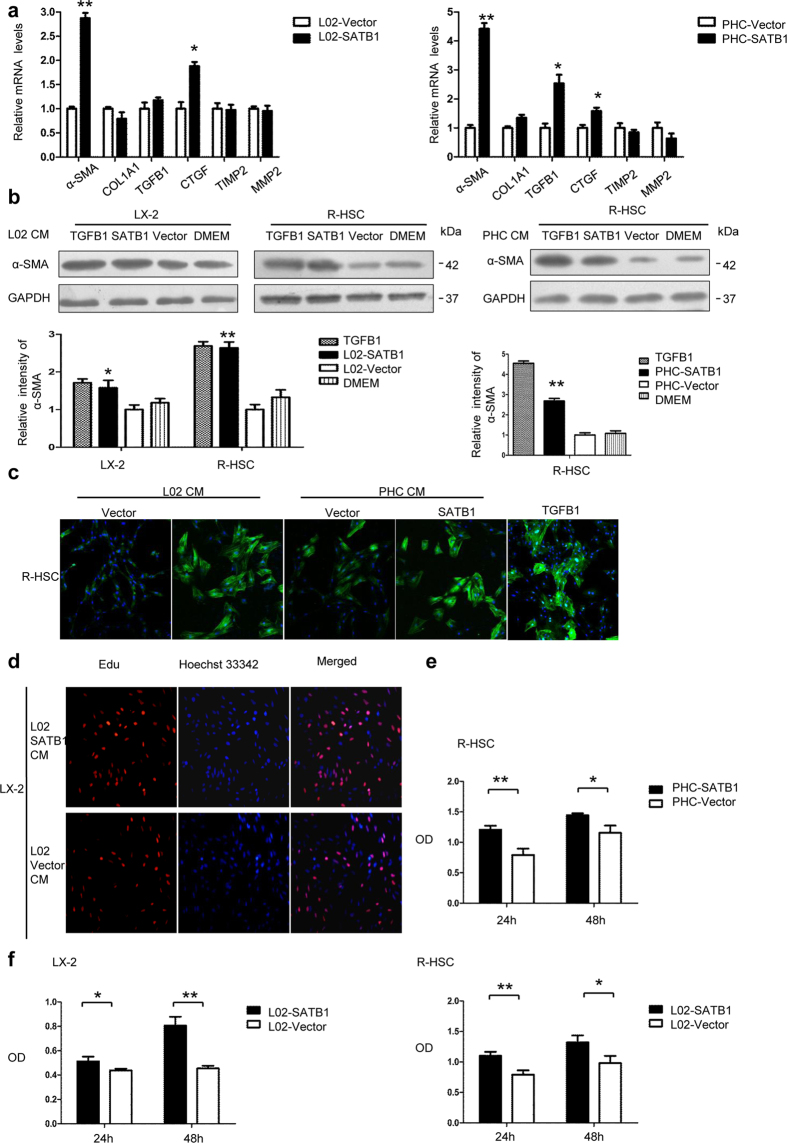
Hepatic SATB1 promotes activation of HSCs. (**a**) LX-2 cells and rat primary stellate cells (R-HSCs) were cultured for 48 h with conditioned medium (CM) from L02 and rat primary hepatic cells (PHC) transduced with lenti-SATB1 and lenti-ctrl virus. The mRNA extracts were used to analyze α-SMA, COL1A1, TGFB1, CTGF, TIMP1, TIMP2 expression. (n = 3). *P < 0.05, **P < 0.01 vs. L02-Vector or PHC-Vector. (**b**) Protein levels of α-SMA were analyzed from LX-2 and R-HSC cells after incubation with CM from lenti-SATB1 or lenti-ctrl expressing L02 cells (L02-SATB1, L02-Vector) and PHC cells (PHC-SATB1, PHC-Vector). *P < 0.05, **P < 0.01 vs. L02-Vector or PHC-Vector. Full-length blots are included in the Supplementary Fig. S9. (**c**) R-HSCs were cultured for 48 h with CM obtained from hepatocytes expressing enhanced SATB1 or control, and α-SMA expression was determined by immunofluorescence analysis. (**d**) LX-2 cell proliferation was detected by EdU assay after incubation with CM from SATB1 expressing L02 cells for 48 h. LX-2 and R-HSCs cell proliferation were determined by cell counting kit-8 (CCK8) after incubation with CM from SATB1 expressing PHC (**e**) and L02 cells (**f**) for 48 h (n = 3). *P < 0.05, **P < 0.01 vs. L02-Vector or PHC-Vector.

It was reported that HBx-expressing hepatocytes activated HSC by TGFB1 in a paracrine way^15^. Having demonstrated HBx upregulated SATB1 levels, we were determined to find out whether hepatic SATB1 could exert a paracrine effect on HSCs. L02 cells and rat primary hepatic cells (PHC) were transduced with lentivectors expressing SATB1 and vectors (Supplementary Fig. S3), culture medium were collected and used to culture human hepatic stellate cell lines LX-2 and rat primary hepatic stellate cells (R-HSCs) for 48 h. We observed that α-SMA was significantly increased in HSCs either incubation with L02-SATB1 or PHC-SATB1 conditioned medium (CM) (Fig. 5a–c). Besides, LX-2 and R-HSCs proliferation was assessed by CCK8 and the rate of incorporation of EdU into the DNA. Notably, SATB1 overexpression in PHC and L02 led to enhanced HSCs proliferation (Fig. 5e,f). In line with this result, the number of LX-2 incorporating EdU was increased when incubation with conditioned medium from L02-SATB1 compared to the control group (Fig. 5d). These observations indicate that SATB1 expressing in liver cells L02 and rat primary hepatic cells can promote the activation and proliferation of HSCs.

### IL-6 and PDGF-AA mediates LX-2 proliferation while CTGF induces paracrine activation of LX-2 by SATB1-overexpressing L02

**Figure 6:**
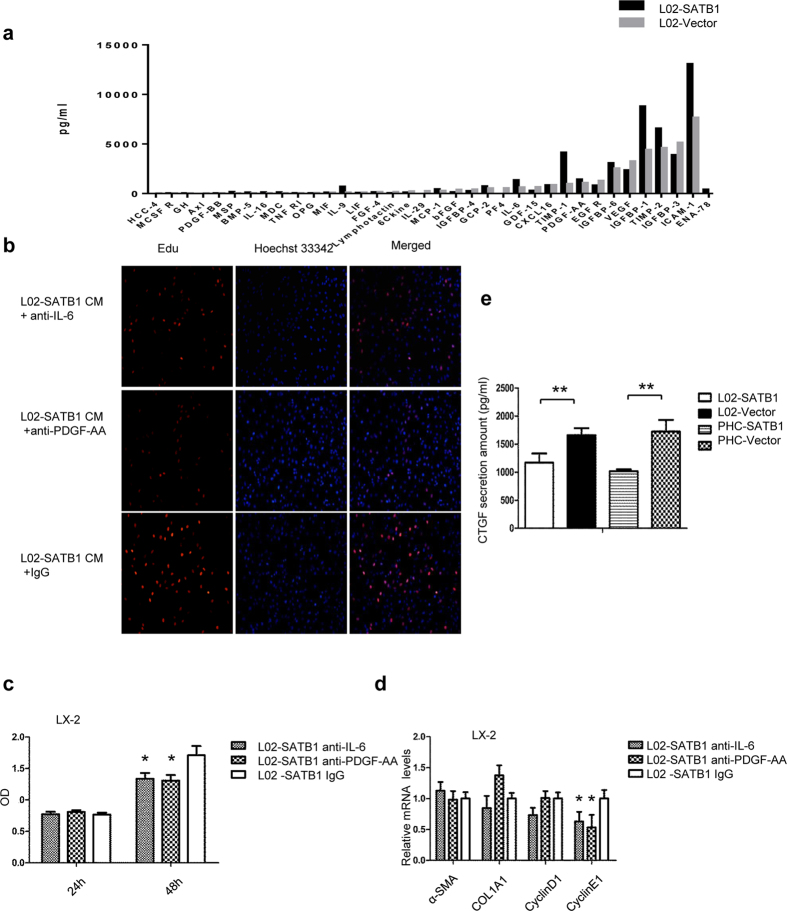
SATB1 activates LX-2 through IL-6, PDGF-AA and CTGF. (**a**) A cytokine profiling was used for the detection of 120 inflammatory factors. Cell supernatant was obtained from L02 cells transduced with lenti-SATB1 and lenti-ctrl for 72 h. (**b,c**) Blocking antibodies against IL-6 (1 ug/ml) and PDGF-AA (1 ug/ml) suppressed L02-SATB1 CM-induced proliferation of LX-2. The results were detected by EdU assay (**b**) and CCK8 (**c**), respectively (n = 3). *P < 0.05, **P < 0.01 vs. L02-SATB1 IgG. (**d**) LX-2 cells were cultured for 48 h with CM obtained from L02 expressing SATB1 (L02-SATB1) in the presence of blocking antibodies IL-6 or PDGF-AA. The mRNA expression of α-SMA, COL1A1, cyclinD1, cyclinE1 were determined by real-time PCR (n = 3). *P < 0.05, **P < 0.01 vs. L02-SATB1 IgG. (**e**) CTGF levels were determined in culture supernatants of L02 and PHC cells infected with lenti-SATB1 or lenti-ctrl for 72 h. (n = 3). *P < 0.05, **P < 0.01 vs. L02-Vector or PHC-Vector.

To identify the factors responsible for HSC activation and proliferation, we performed a cytokine profile of CM obtained from L02 cells overexpressing SATB1 and control group. TIMP1, TIMP2, IL-6, PDGF-AA were detected at high levels and significantly elevated in conditioned media of L02-SATB1 compared to the controls (Fig. 6a). Therefore, we incubated LX-2 with conditioned media from L02-SATB1 in the presence of blocking antibodies against IL-6, PDGF-AA. We found that incubation of HSCs with blocking antibody against IL-6 and PDGF-AA abolished the increase in the proliferation induced by CM from L02-SATB1, while neutralizing antibodies against IgG showed no effect on LX-2 proliferation (Fig. 6b,c). However, the blocking antibody against IL-6, PDGF-AA both showed no effect on the increase in α-SMA expression in LX-2 in the presence of supernatants from L02-SATB1 (Fig. 6d). It has been reported SATB1 expression induced a marked change in many genes associated with metastasis in cancer cells, transforming growth factor-β1 (TGFB1) and connective tissue growth factor (CTGF) included^23^. Since both of them are responsible for HSCs activation, we analyzed the secretion of TGFB1 and CTGF from conditioned medium of SATB1 expressing L02 and PHC. The results of cytokine profile showed no prominent difference of TGFB1 between L02-SATB1 and control group (Supplementary Table S3). Moreover, L02-SATB1 and PHC-SATB1 induced CTGF secretion, when compared to the control croup respectively (Fig. 6e). Altogether, these findings demonstrate that IL-6 and PDGF-AA secreted by L02-SATB1 are responsible for enhanced proliferation of LX-2, while CTGF may mediates the paracrine activation of HSCs by SATB1-expressing liver cells.

### SATB1 involves in HBx-mediated paracrine activation of HSCs

**Figure 7:**
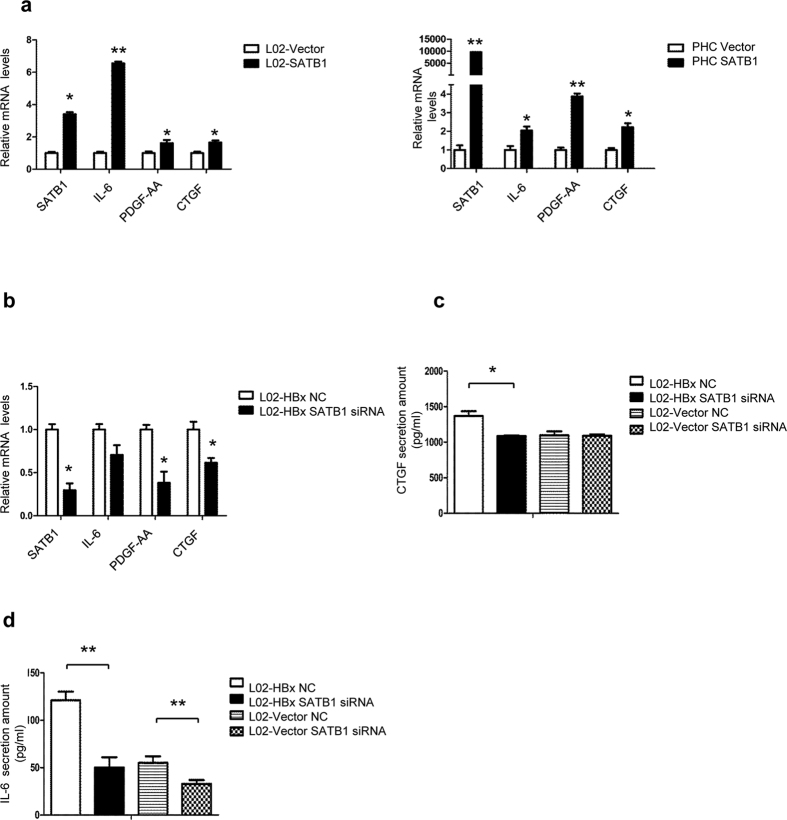
SATB1 participates in HBX-mediated fibrogenesis by modulation of IL-6, PDGF-AA and CTGF expression. (**a**) The mRNA levels of SATB1, IL-6, PDGF-AA and CTGF were analyzed by Real-time PCR in L02 and PHC cells transduced with lenti-SATB1 and lenti-ctrl virus. (n = 3). *P < 0.05, **P < 0.01 vs. L02-Vector or PHC-Vector. (**b**) Silencing SATB1 in L02-HBx cells for 48 h, mRNA extracts were used for analyzing SATB1, IL-6, PDGF-AA, and CTGF expression by Real-time PCR. (n = 3). *P < 0.05, **P < 0.01 vs. L02-HBx NC. (**c,d**) Silencing SATB1 expression in L02-HBx cells for 48 h, the conditioned medium were used to determine the secretion amount of IL-6 and CTGF. (n = 3). *P < 0.05, **P < 0.01 vs. L02-HBx NC or L02-Vector NC.

We have showed that SATB1, as the downstream of HBx, responsible for the paracrine activation of LX-2 by secretion of IL-6, PDGF-AA and CTGF (Fig. 7a). Next, we analyzed the role of SATB1 in HBx-mediated activation of HSCs. Silencing SATB1 in HBx-transfected cells could down-regulated the mRNA levels of PDGF-AA and CTGF (Fig. 7b). Besides, knockdown of SATB1 decreased the secretion of IL-6 and CTGF in HBx-transfected L02 cells (Fig. 7c,d). All together, these results suggest that SATB1 involves in HBx-mediated HSCs activation by regulation of cytokines IL-6, PDGF-AA and CTGF.

## Discussion

Accumulating studies have demonstrated that the activation of hepatic stellate cells (HSCs) is a hallmark of hepatic fibrogenesis. However, the role of hepatocytes in liver fibrosis was lack of investigation in the past decades. In this work, we provided different lines of evidence supporting a direct causal link between SATB1 activation in hepatocytes and liver fibrosis. First, hepatic SATB1 expression is positively upregulated in patients and mouse model with HBV-related fibrosis. Second, inhibiting SATB1 expression alleviates liver fibrogenesis in the HBV transgenic mouse model. Third, SATB1 overexpression induces paracrine activation of HSCs by secretion of inflammatory cytokines IL-6, PDGF-AA and CTGF. We have previously shown high expression of SATB1 in HCC could potentiate cancer cell with enhanced proliferating and metastatic ability^27^. In line with the present study, we identified the expression of SATB1 in hepatocytes obviously increased during the progression of fibrosis and hepatocellular carcinoma. However, our previous work demonstrated SATB1 plays an antifibrotic effect in TAA-induced rat model, because SATB1 inhibits HSC activation, proliferation, migration and collagen synthesis^29^. These studies indicated the pathogenic role of SATB1 in liver disease remains contradictory, partly because different cell types endowed SATB1 with different function. Our former data reported SATB1 repressed CTGF expression in HSCs, whereas contrasting results revealed SATB1 overexpression promotes CTGF expression, cell proliferation and migration in some cancer cell lines and hepatocytes^23^. Additionally, it was reported SATB1 also had opposite effect on cell proliferation between mouse embryonic fibroblasts (MEFs) and tumor cells^35^. Another reason to explain the controversial results is the different models that were used and various liver pathogenic conditions. SATB1 exerts antifibrotic role in the model of TAA-induced fibrosis, because positive staining of SATB1 was only seen in the nucleus of liver non-parechymal cells. Our current work studied liver fibrogenesis in HBV transgenic (HBV-Tg) mice to partly mimic liver fibrotic progression during chronic HBV infection^30^. In agreement with our clinical data, hepatic SATB1 expression was positively associated with HBV-related fibrosis and mainly expressed in hepatocytes. Besides, SATB1 can trigger the activation of HSCs by release of pro-inflammatory signals and proliferation-associated cytokines. Another study reported the same case indicated that the ultimate effect of IL-22 in liver fibrosis depends on its effect on hepatocyte protection and liver inflammatory damage^18^. Therefore, distinct pathological basis and effector cells may determine the action of SATB1 in liver fibrosis.

Recently, several studies suggested there existed crosstalk between hepatocytes and HSC, such as overexpression of c-Myc, p53 in hepatocytes contributed to HSC activation^36^,^37^, yet hepatic HNF1a showed antifibrotic function. However, the role of hepatocytes in the activation of HSCs during chronic HBV infection progressed into liver cirrhosis has not been clearly elucidated. It was reported the HBx promoted liver fibrosis through a paracrine action on HSCs activation and proliferation. Several studies have reported expression of HBx in hepatic cells led to production of interleukin-6 (IL-6), CTGF and TGFB1^15^,^16^,^38^. Our experiments show that SATB1 might participate in the HBx-mediated profibrotic process, because silenced SATB1 in HBx-expressing L02 inhibited the expression of profibrotic factors CTGF, IL-6 and PDGF-AA. Our data also confirmed HBx might activate JNK and ERK pathways, subsequently modulating SATB1 expression via AP-1. These findings demonstrated that SATB1 may function as an important medium in HBV-induced liver fibrosis.

HSCs and intrahepatic immune cells are regarded as the principal source of production of inflammatory cytokines during chronic liver injury^39^. Actually, hepatocytes actively engage in cross-talk with multiple cellular subsets, along with the release of reactive oxygen species (ROS) and pro-inflammatory signals^3^. Here we show that overexpresssion of SATB1 in hepatocytes led to upregulation of CTGF, IL-6 and PDGF-AA. CTGF, mainly produced by activated HSCs, function as an important profibrogenic cytokine that could drive HSC activation and liver fibrogenesis. Recent studies show that CTGF also could be produced from hepatocytes. This finding supports our result that enhanced expression of hepatic SATB1 mediated the synthesis of CTGF. PDGF-A and PDGF-B and their receptors are expressed in liver myofibroblasts, PDGFs could stimulate the migration and proliferation of stellate cell, mediated inflammation and fibrogenesis during the liver injury^40^,^41^. In addition, HBx induced IL-6 production in hepatocytes and hepatoma cells, IL-6 could trigger hepatic cells proliferation and liver regeneration, and modulate synthesis of collagen I^42^. In this work, we found cocultured LX-2 showed declined proliferating ability when inhibited by neutralizing anti-IL-6, anti-PDGF-AA antibody. These results indicated SATB1 promoted LX-2 proliferation partly through modulation of IL-6 and PDGF-AA. However, the screening range of our cytokine profile was limited, whether there existed other profibrotic factors are not determined. In the present study, we unveiled IL-6, PDGF-AA, CTGF could be the crucial factors contributed to SATB1-mediated HSCs activation.

In conclusion, we demonstrated hepatic SATB1 promotes paracrine activation of HSCs in HBV-related fibrosis. These findings highlight the significance of SATB1 and hepatocytes in liver fibrogenesis and elucidated the role of SATB1 in HBV-related chronic liver disease.

## Methods

### Cell Lines, Primary cells isolation, and culture

We grew the hepatic cell line L02 and epithelial cell line originating from HeLa cells Chang Liver (CHL) in Dulbecco’s modified Eagle’s medium (DMEM) with 10% fetal bovine serum (FBS). Chang Liver results were indicated as non-hepatic control. Rat primary hepatic cells (PHC) and rat primary hepatic stellate cells (R-HSCs) were isolated from male Sprague-Dawley (SD) rats, and cultured in DMEM with 20% FBS, penicillin and streptomycin as previously described^29^, and the primary HSCs were used for up to four passages. For the expriments, we plated SATB1 expressing hepatocytes at a density of 100,000 cells/cm^2^ in DMEM 10% FBS and shifted to DMEM without FBS 24 hours later. The cultured mediums were collected 48 h later, and stored in cryovials at −80 °C until use.

### Patients, liver samples and HE staining

Following ethical and institutional guidelines and after informed consent of the tissue donors, a total number of 68 human liver samples were collected from surgical resections of patients in Tongji Hoptital (Wuhan, China). Detailed clinical pathologic information was listed in Supplementary Table S1. The procedure of human sample collection was approved by the Ethics Committee of Tongji Hospital, Huazhong University of Science and Technology and the study was conducted according to the Declaration of Helsinki principles. HE staining, the HAI scoring system was used to assess the severity of liver fibrosis.

### Virus

Adenoviral vector containing shRNA targeting SATB1 (AdshSATB1) and the control adenovirus (AdshNC) were purchased from Genechem Co. Ltd. (Shanghai, China). PcDNA3.1-SATB1 plasmids or pcDNA3.1 control vector were previously established in our lab. Lentiviral vectors containing SATB1 (lenti-SATB1) and the control (lenti-ctrl) were packaged from Genechem Co. Ltd. (Shanghai, China).

### Animals and treatment

HBV transgenic mice C57BL/6J-TgN(AlblHBV) 44Bri containing HBV genome S, pre-S and X domains, were purchased from Peking University Health Science Center (Beijing, China). Male 7 to 10-week-old transgenic mice (HBV-Tg mice) were intraperitoneally (0.5 uL/g of body weight, twice a week) injected with 25% CCl4 to induce chronic liver injury. Several weeks’ injection (0, 4 and 8 weeks, respectively) were made to establish fibrosis with different degree of severity. To observe the effect of SATB1 on hepatic fibrosis, 5 × 10^8^ pfu AdshNC or Ad shSATB1 was injected via tail vein once a week for 2 weeks at the beginning of the 6th week’s CCl4 injection, and mice were sacrificed 72 hours following the last CCl4 injection, and liver tissues and serum were collected. All animal experiments were in accordance with the National Institute of Health Guide for the Care and Use of Laboratory Animals, and were approved by the Committee on the Ethics of Animal Experiments of Tongji Medical College.

### Co-culture of HSCs and hepatocytes

Liver cell lines L02 and rat primary hepatic cells were infected with lenti-SATB1 or lenti-ctrl at a MOI of 10 for 12 h, and then the cells cultured with 1 ng/ml puromycin. Hepatic cell ovexpressing SATB1 and the control group were co-cultured with human HSCs cell lines, LX-2, and primary rat HSCs for 48 h in the upper-chamber of 0.4 um trans-well plates (3450, Corning). Antibodies against IL-6, PDGF-AA (R&D System) or an isotype-matched negative control were added into the medium to neutralize the profibrotic factors. Total RNA of HSCs lysate were harvested 48 h later.

### Transient RNA interference

Small interfering RNA (siRNA) duplexes targeting human SATB1, c-Jun, NFkB1, Sp1 sequence and scrambled siRNA were produced by Ribobio Co. Ltd. (Guangzhou, China) and their knock-down efficiency was validated by Real-time PCR and Western Blot. siRNA sequences were as follows:

SATB1, sense, 5′-GGAUAGUCUUUCUGAGCUA-3′, antisense, 5′-UAGCUCAGAAAG ACUAUCC-3′;

Sp1 si-1, sense, 5′-CCAACAGAUUAUCACAAAU-3′, antisense, 5′-AUUUGUGAUAAUCUGUUGG-3′;

Sp1 si-2, sense, 5′-GGCUGGUGGUGA UGGAAUA-3′, antisense, 5′-UAUUCCAUCACCACCAGCC-3′;

NFKB1, sense, 5′-GGGGCUAUAAUCCUGGACU-3′, antisense, 5′-AGUCCAGGAUUGUAGCCCC-3′;

c-Jun, sense, 5′-CUGCAAAGAUGGAAACGAC-3′, antisense, 5′-GUCGUUUCCAUCUUUGCAG-3′,

NC, sense, 5′-UUCUUCGAACGUGUCAC G-3′, antisense, 5′-ACGUGACACGUUCGGAGAATT-3′.

### Western blotting and Enzyme-Linked Immunoabsorbent Assay

Cell extracts and live tissues were digested in 1x RIPA buffer containing phosphatase inhibitor and PMSF (Goodbio Technology Co. Ltd. Wuhan, China). For western blot, the membrane was incubated overnight at 4 °C with primary antibodies: anti-p38 (ab195049, Abcam); anti-c-Jun (9165p, Cell Signaling Technology); anti-α-SMA (BM0002, Boster); anti-SATB1(3650, Cell Signaling Technology); anti-Sp1(10915, santa cruz); anti-Akt (21054, signalway antibody); anti-phospho-Akt (11054, signalway antibody); anti-ERK1/2 (1016, Cell Signaling Technology); anti-phospho-ERK1/2 (9101, Cell Signaling Technology), anti-phospho-p38 (11581, signalway antibody); anti-rabbit IgG-HRP (Promoter Biotechnology Ltd, China); anti-NFKB1 (ab32360, Abcam). Then followed by anti-mouse or -rabbit IgG (1:3,000; Sigma, CA), and the signals were detected with an ECL assay kit (Amersham, Buckinghamshire, UK). IL-6 (EHC007, NeoBioscience) and CTGF (900-K317, peprotech) enzyme-linked immunobsorbent assay (ELISA) were performed following the instructions by the manufacturer.

### Immunohistochemistry analysis

Masson staining was used for collagen (Goodbio Technology Co. Ltd. Wuhan, China). Immunohistochemistry was performed on paraffin-embedded liver sections. Antibodies against SATB1 (Ab109122, Abcam), α-SMA (BM0002, Boster, Wuhan, China) were used for immunohistochemistry. The overall score of each section was assessed by multiplying the intensity score by percentage score of positive staining cells. The classification of each section was determined by the scoring system previously described^43^.

### HSC proliferation measurement

we seeded 4000/well HSCs in 96 well plates. And incubated them in conditioned medium from SATB1 overexpressing hepatocytes and the control group for 24 and 48 h, respectively. The results confirmed by CCK8 (Promoter Biotechnology, Wuhan, China) and EdU kit (Riobio Co. Ltd. Guangzhou, China).

### Cytokines profile, and RT-PCR

Secretion profiles of lenti-SATB1 and lenti-ctrl transduced L02 cells were determined using human cytokine antibody arrays (QAH-CAA-2000; Raybiotech, Inc.). The result of human cytokine antibody arrays between lenti-SATB1 and lenti-ctrl transduced L02 cells was listed in Supplementary Table 3. Total RNA was extracted using TRIzol (Takara, Otsu, Japan) and cNDA was synthesized using the PrimeScript RT reagent kit (Takara, Otsu, Japan). Real-time polymerase chain reaction was performed using SYBR Premix ExTaq (Takara, Otsu, Japan) on an ABI StepOne Real-Time PCR System (Applied Biosystem, Carlsbad, CA,USA). The value of 2–ΔΔCt was used to determine fold difference between samples. The sequences of the primers used for PCR are listed in Supplementary Table S2.

### Immunofluorenscence staining

Cells fixed with 4% PFA solution, permeabilied with Tritonx100 were incubated with an anti-α-SMA antibody overnight, after with Alexa Fluor 488 conjugated anti-mouse secondary antibody (Promoter biotechnology, Wuhan, China), DAPI was used for nuclear staining. The final sections were examined by Confocal laser scanning microscope.

### Luciferase reporter experiments

SATB1 reporter (−1087 bp/+168 bp) activity was detected by Secrete-Pair^TM^ Dual Luminescence Assay Kit (GeneCopoeia) according to the manufacturer’s instructions.

### Statistical analysis

All experiments were performed in triplicate. The data are presented as mean ± SD. Statistical analyses were performed by Student’s t-test. The pearson chi-square was used to analyze the correlation between SATB1 expression and pathogenic conditions of chronic liver disease in human sample. Statistical analysis was performed with Prism 5.0 (GraphPad Software, La Jolla, CA, USA). A value of p < 0.05 was considered statistically significant.

## Additional Information

**How to cite this article**: Gong, J. *et al*. Hepatic SATB1 induces paracrine activation of hepatic stellate cells and is upregulated by HBx. *Sci. Rep.* **6**, 37717; doi: 10.1038/srep37717 (2016).

**Publisher's note:** Springer Nature remains neutral with regard to jurisdictional claims in published maps and institutional affiliations.

## Supplementary Material

Supplementary Information

## Figures and Tables

**Table 1:** SATB1 protein expression in each group.

Group	HC (n = 13)	CHB (n = 30)	LC (n = 25)
SATB1 positive(n, %)	2	15	13
SATB1 negative(n, %)	11	15	12
*P* value		#*P* = 0.033	*P = 0.028

Note: ^#^HC VS CHB; ^*^HC vs LC: pearson chi-square.
