# Author Correction: Hepatic SATB1 induces paracrine activation of hepatic stellate cells and is upregulated by HBx

**DOI:** 10.1038/s41598-021-87154-6

**Published:** 2021-03-31

**Authors:** Jin Gong, Wei Tu, Jian Han, Jiayi He, Jingmei Liu, Ping Han, Yunwu Wang, Mengke Li, Mei Liu, Jiazhi Liao, Dean Tian

**Affiliations:** 1 Department of Gastroenterology, Tongji Hospital of Tongji Medical College, Huazhong University of Science and Technology, Wuhan, 430030 China; 2 Department of PediatricsTongji Hospital of Tongji Medical College, Huazhong University of Science and Technology, Wuhan, 430030 China

Correction to: *Scientific Reports* https://doi.org/10.1038/srep37717, published online 24 November 2016

This Article contains errors. As a result of a figure assembly error, in Figure 4a the representative blot for SATB1 of CHL-HBx is a duplication of L02-HBx. The correct Figure 4 appears below as Figure 1.

**Figure 1:**
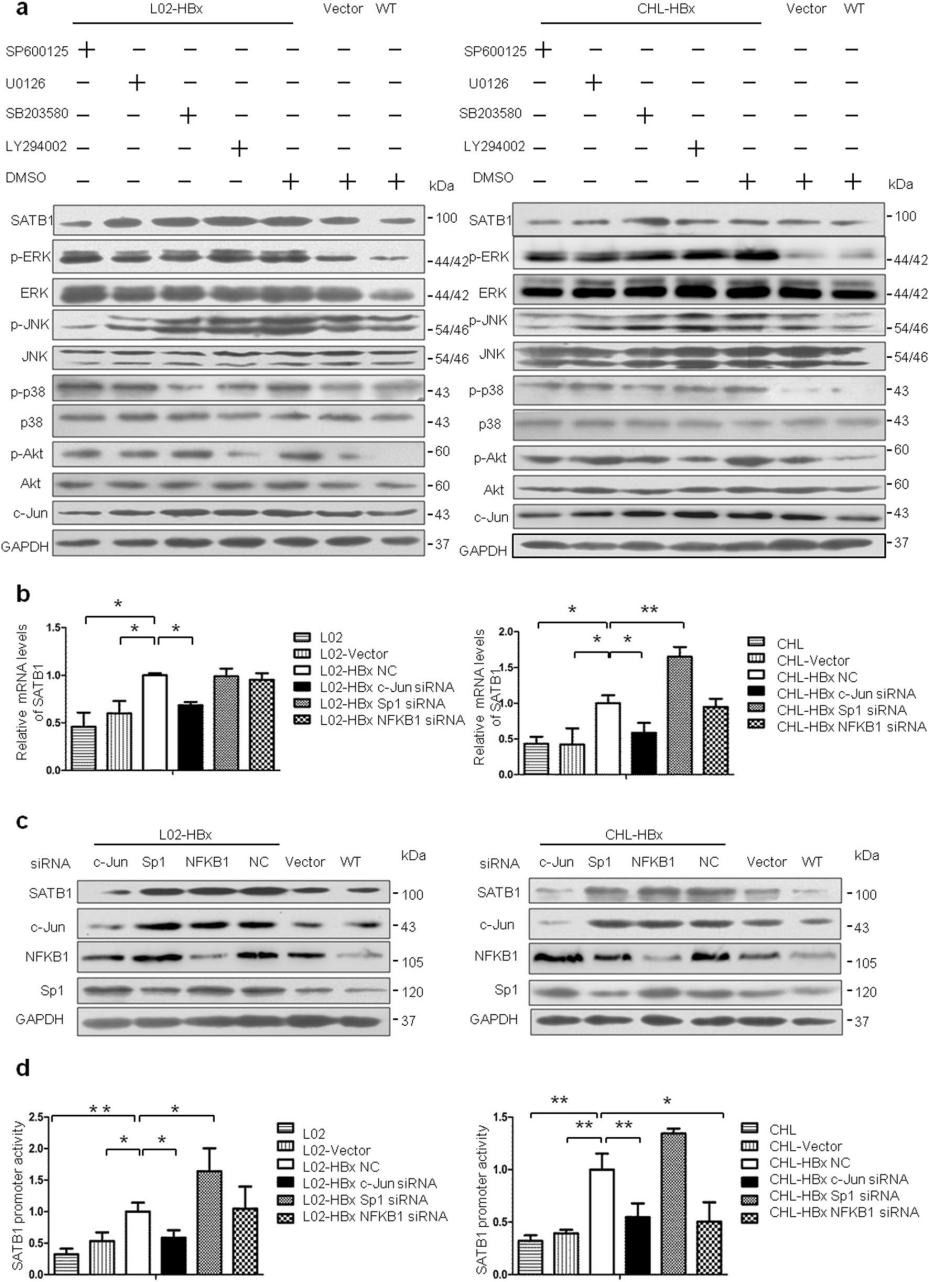
A correct version of the original Figure 4.

