# Supplementary material for: Hepatic SATB1 induces paracrine activation of hepatic stellate cells and is upregulated by HBx
Source: Sci Rep. 2016 Nov 24;6:37717. doi: 10.1038/srep37717 (PMC5121621; doi:10.1038/srep37717)
Supplement: Supplementary Information [file srep37717-s1.pdf]

Supplementary data to:

Hepatic SATB1 induces paracrine activation of hepatic stellate cells and is upregulated by HBx

Jin Gong[1#], Wei Tu[1#], Jian Han[1], Jiayi He[2], Jingmei Liu[1], Ping Han[1], Yunwu Wang[1], Mengke Li[1], Mei Liu[1], Jiazhi Liao[1],Dean Tian[1 *].

[1]Department of Gastroenterology, Tongji Hospital of Tongji Medical College, Huazhong University of Science and Technology, Wuhan, Hubei Province, China

[2]Department of Pediatrics, Tongji Hospital of Tongji Medical College, Huazhong University of Science and Technology, Wuhan, Hubei Province, China

Supplementary Figures

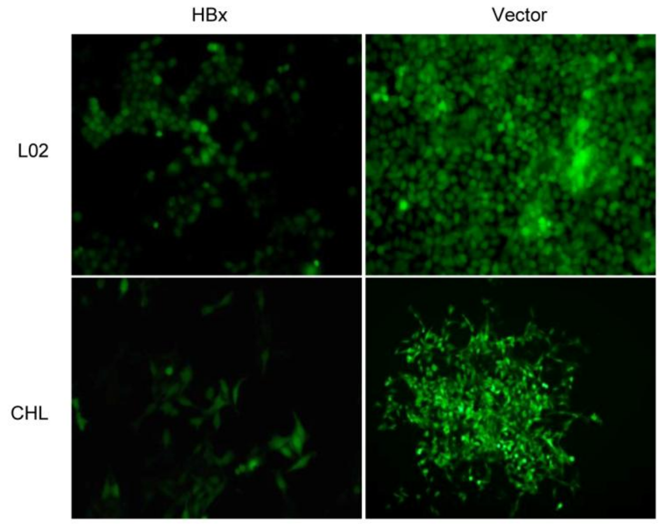

Supplementary Fig .S1. Stable transfection of PEGFP-N1-HBX and PRGFP-N1-Vector plasmid into L02 (L02-HBx) and Chang liver cells (CHL-HBx), cells were analyzed under green fluorescent field (right) (100x) .

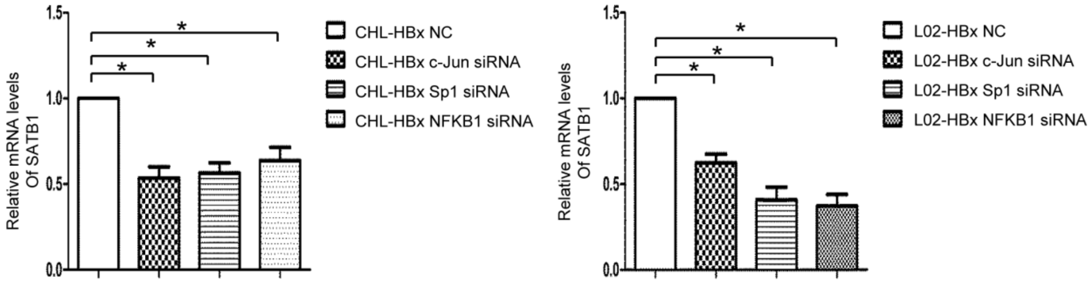

Supplementary Fig .S2. C-jun, Sp1, NFKB1 and NC control siRNA were transfected into L02-HBx and CHL-HBx, respectively. Real-time PCR was used to analyze the mRNA levels of SATB1 expression 48h later.

a.

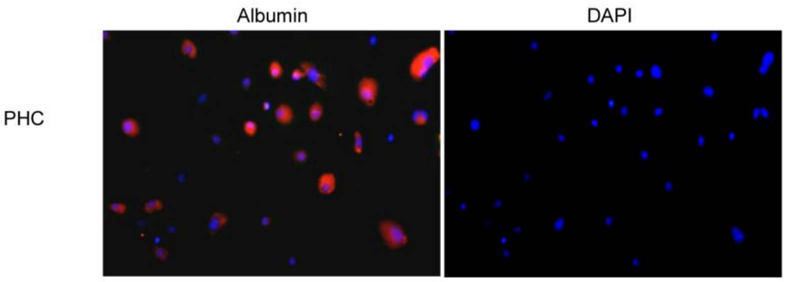

b.

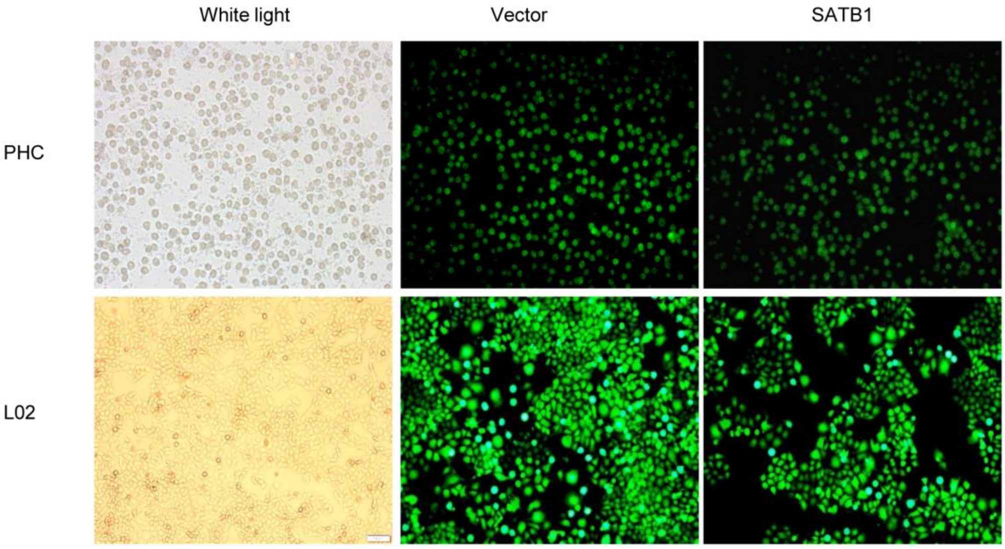

Supplementary Fig .S3. (a) Identification of freshly isolated primary hepatic cells (PHC) from rats. Albumin expression was determined by immunofluorescence analysis. (b) Hepatic cell line L02 and rat primary hepatic cells (PHC)were transduced with lenti-SATB1 and lenti-ctrl virus, respectively. Cells were analyzed under white light field and green fluorescent field (100x).

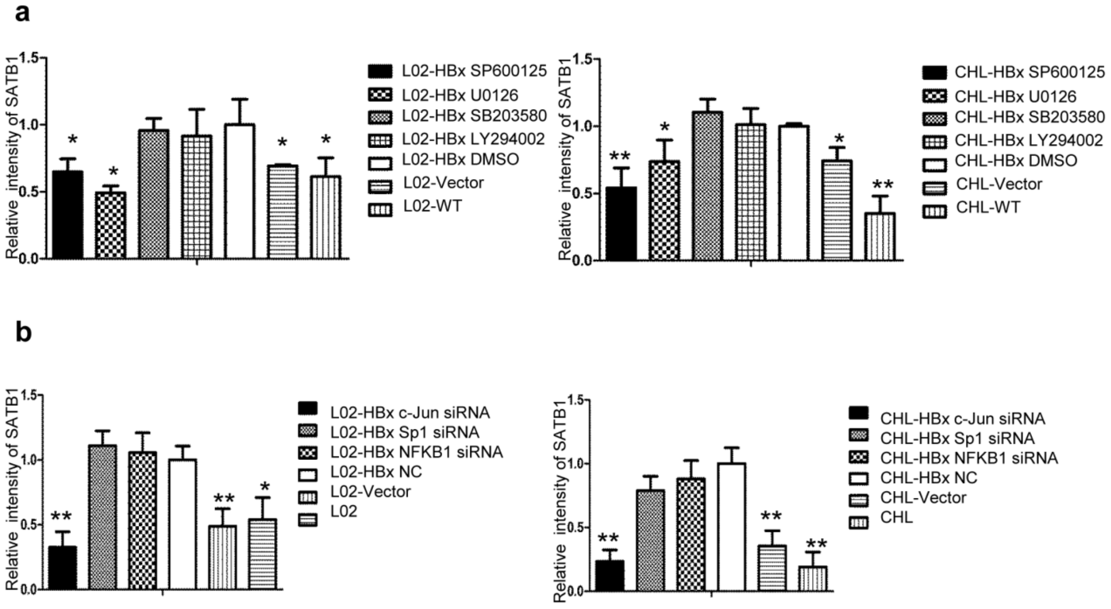

Supplementary Fig .S4. The protein expression were measured by western blot. Image J software were used to analyze protein gray scale in WB. Relative intensity of protein levels were presented as column chart. *P < 0.05, **P < 0.01 vs. HBx DMSO or HBx NC.

Supplementary Fig .S5. full-length blots of Fig.1d are included in the Supplementary Information file.

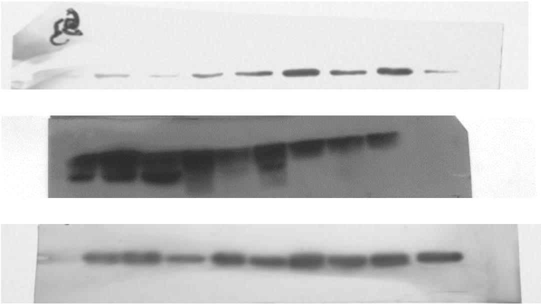

SATB1(100 kDa)

α-SMA (42 kDa)

GAPDH (37 kDa)

Supplementary Fig .S6. full-length blots of Fig.2b are included in the Supplementary Information file.

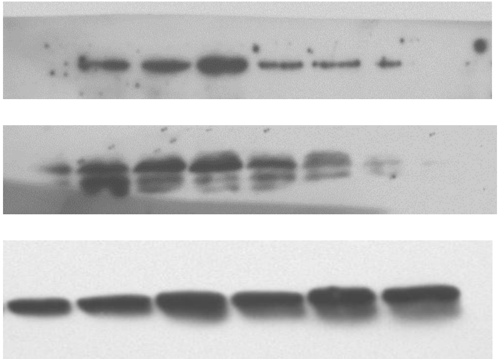

SATB1 (100 kDa)

α-SMA (42 kDa)

GAPDH (37 kDa)

Supplementary Fig .S7. full-length gels and blots of Fig.3 are included in the Supplementary Information file.

a

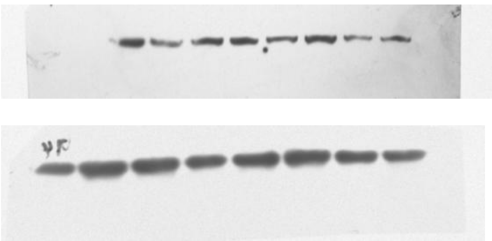

SATB1 (100 kDa)

GAPDH (37 kDa)

b

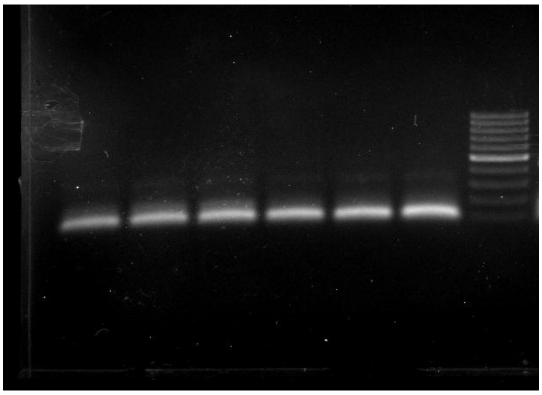

GAPDH

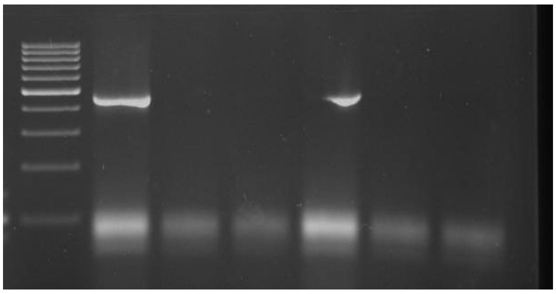

HBx

d.

L02 cell line

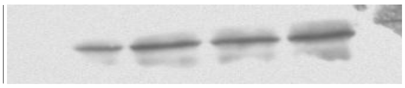

SATB1(100 kDa)

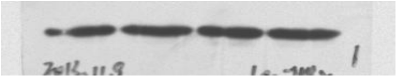

GAPDH (37 kDa)

Chang liver cell line

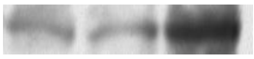

SATB1 (100 kDa)

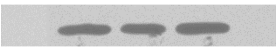

GAPDH (37 kDa)

Supplementary Fig .S8. full-length gels and blots of Fig.4 are included in the Supplementary Information file.

L02 cell line

a

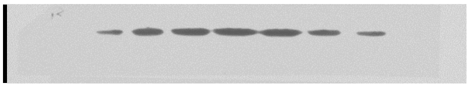

SATB1 (100 kDa)

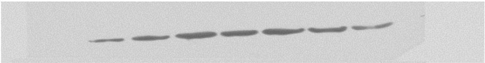

c-Jun (43 kDa)

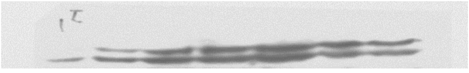

p-JNK (54/46 kDa)

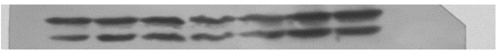

JNK （54/46 kDa））

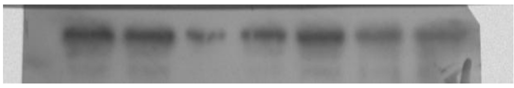

p-p38 (43 kDa)

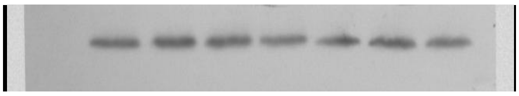

P38 (43 kDa)

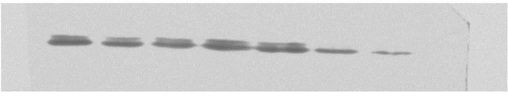

p-ERK (44/42 kDa)

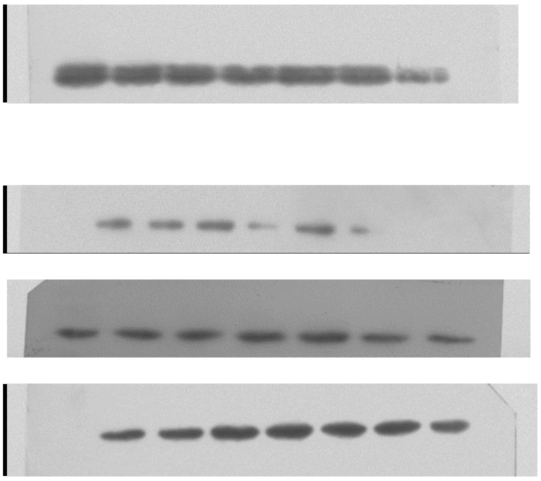

ERK (44/42 kDa)

p-Akt (60 kDa)

Akt (60 kDa)

GAPDH (37 kDa)

Chang liver cell line

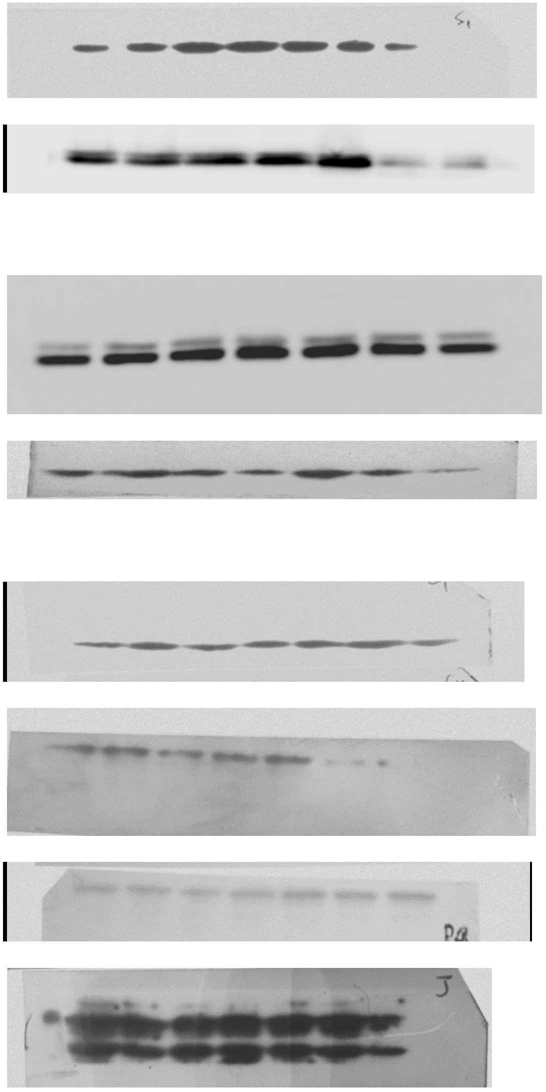

SATB1 (100 kDa)

p-ERK (44/42 kDa)

ERK (44/42 kDa)

P-Akt (60 kDa)

Akt (60 kDa)

P-p38 (43 kDa)

P38 (43 kDa)

JNK (54/46 kDa)

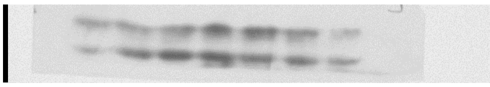

p-JNK (54/46 kDa)

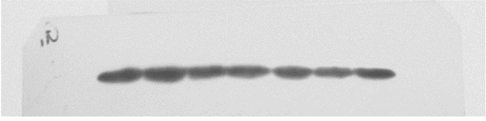

GAPDH (37 kDa)

c

L02 cell line

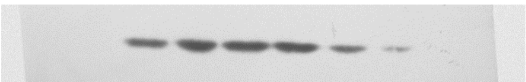

SATB1 (100 kDa)

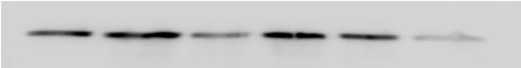

NFKB1 (105 kDa)

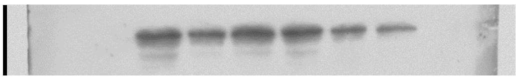

Sp1 (120 kDa)

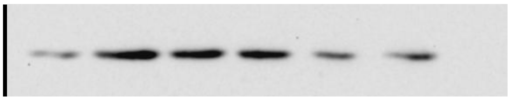

c-Jun (43 kDa)

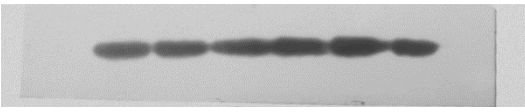

GAPDH (37 kDa)

Chang liver cell line

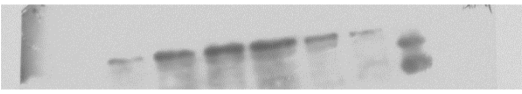

SATB1 (100 kDa)

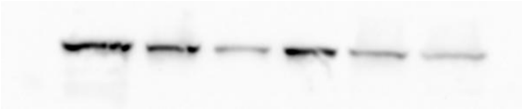

NFKB1 (105 kDa)

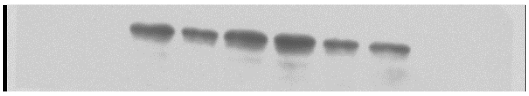

Sp1 (120 kDa)

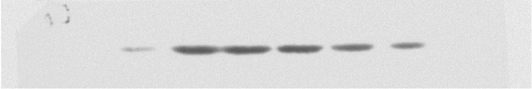

c-Jun (43 kDa)

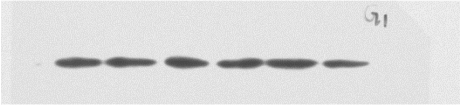

GAPDH (37 kDa)

Supplementary Fig .S9. full-length blots of Fig.5b are included in the Supplementary Information file.

L02 CM+LX-2

b

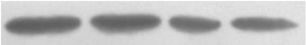

a-SMA (42 kDa)

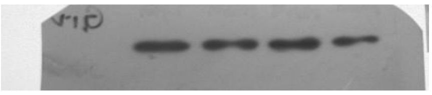

GAPDH (37 kDa)

L02 CM+R-HSC

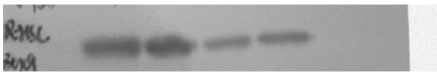

a-SMA (42 kDa)

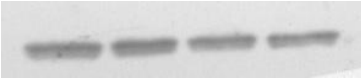

GAPDH (37 kDa)

PHC CM+R-HSC

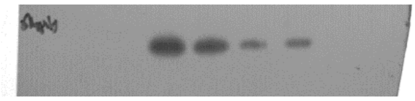

a-SMA (42 kDa)

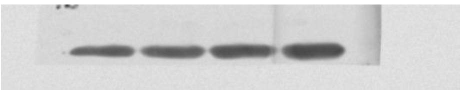

GAPDH (37 kDa)

Supplementary Tables

Supplementary Table S1. Clinical Charateristics of Enrolled Subjects

**Category**	**HC**	**CHB**	**LC**
cases	13	30	25
Ages,years	43.8	44.4	44.2
Gender,M/F	7/6	12/18	16/9
ALT,IU/L	20.9	19.9	20.1
AST,IU/L	21.9	17.9	24.2
Serum albumin,g/L	38.5	42.1	36.4
Total bilirubin,umol/L	9.9	9.2	21.7
Direct bilirubin,umol/L	3.2	3.5	9.7
Prothrombin activity,%	92.3	103.3	63.5
HBeAg,positive/negative	0/13	0/30	3/22
Serum HBV levels,IU/mL	ND	4.0×10^3	5.6×10^4

Abbreviations: M, male; F, female; HBeAg, hepatitis B e antigen;ND, not determined.

Supplementary Table S2. Primers for quantitative real-time PCR.

**primer**	**Forward primer sequence**（5’-3’）	**Reverse primer sequence**（5’-3’）
**human**		
SATB1	GATCATTTGAACGAGGCAACTCA	TGGACCCTTCGGATCACTCA
IL-6	ACTCACCTCTTCAGAACGAATTG	CCATCTTTGGAAGGTTCAGGTTG
CTGF	CAGCATGGACGTTCGTCTG	AACCACGGTTTGGTCCTTGG
PDGF-AA	CCCATTCGGAGGAAGAGAAG	ATCAGGAAGTTGGCGGACG
GAPDH	ACAACTTTGGTATCGTGGAAGG	GCCATCACGCCACAGTTTC
COL1A1	CCCCTGGAAAGAATGGAGAT	AATCCTCGAGCACCCTGA
α-SMA	ACTGCCGCATCCTCATCC	ATGCTGTTGTAGGTGGTTTCAT
TGFB1	GGAAATTGAGGGCTTTCGCC	CGGTAGTGAACCCGTTGATG

TIMP2	AAGCGGTCAGTGAGAAGGAAG	GGGGCCGTGTAGATAAACTCTAT
MMP2	CCA GAT GTG GCC AAC TAC AA	GGT CAG GTG TGT AAC CAA TGA
c-Jun	TCCAAGTGCCGAAAAAGGAAG	CGAGTTCTGAGCTTTCAAGGT
NFKB1	AACAGAGAGGATTTCGTTTCCG	TTTGACCTGAGGGTAAGACTTCT
Sp1	GTGGCCGCTACCTTCACTG	GCCCCACTCCTACTTGGTC
cyclinE1	AAGGAGCGGGACACCATGA	ACGGTCACGTTTGCCTTCC
cyclinD1	GCTGCGAAGTGGAAACCATC	CCTCCTTCTGCACACATTTGAA
**mouse**		
SATB1	ATGGATCATTTGAACGAGGCAA	GACCCTTCGGATCACTCACA
IL-6	TAGTCCTTCCTACCCCAATTTCC	TTGGTCCTTAGCCACTCCTTC
CTGF	GACCCAACTATGATGCGAGCC	CCCATCCCACAGGTCTTAGAAC
GAPDH	AGGTCGGTGTGAACGGATTTG	TGTAGACCATGTAGTTGAGGTCA
COL1A1	AGAGGCGAAGGCAACAGTCG	GCAGGGCCAATGTCTAGTCC
α-SMA	CCCAGACATCAGGGAGTAATGG	TCTATCGGATACTTCAGCGTCA
**rat**		
SATB1	ACGCCTCCATTTATGACGA	CTCCCACAGGGTTCTGTTTT
α-SMA	CCCAGACATCAGGGAGTAATGG	TCTATCGGATACTTCAGCGTCA
COL1A1	AGAGGCGAAGGCAACAGTCG	GCAGGGCCAATGTCTAGTCC
TIMP2	TCAGAGCCAAAGCAGTGAGC	GCCGTGTAGATAAACTCGATGTC
TGFB1	CTTCAATACGTCAGACATTCGGG	GTAACGCCAGGAATTGTTGCTA
IL-6	CCCACCAGGAACGAAAGTCA	ACTGGCTGGAAGTCTCTTGC
PDGF-A	GAACCGCAAAGAGGTGTCCT	TTGTTCTACGCGTCCTGTCC
GAPDH	AGGTCGGTGTGAACGGATTTG	TGTAGACCATGTAGTTGAGGTCA

Supplementary Table S3. Results of human cytokine antibody arrays (QAH-CAA-2000) were analyzed between lenti-SATB1 and lenti-ctrl transduced L02 cells.

(pg/ml)	**L02-SATB1**	**L02-Vector**
IL-9	689.3	100.3
MIG	54.86	0
IGFBP-1	8,788.6	4,411.6

PDGF-AA	1,424.8	1,067.9
ICAM-1	13,074.0	7,654.8
IL-1a	31.8	6.5
IL-6	1,340.8	621.9
VEGF-D	26.72	15.17
IL-15	29.4	0.0
IL-16	117.1	40.5
MCP-1	434.1	262.7
MCSF	41.5	0.0
TIMP-1	4,133.7	969.6
TIMP-2	6,557.6	4,594.1
bFGF	136.5	386.9
EGF R	802.4	1,276.9
GDF-15	282.1	642.5
IGFBP-3	3,877.2	5,132.6
VEGF	2,345.1	3,247.3
CXCL16	833.0	854.4

IGFBP-6	3,072.5	2,527.6
TGFB1	0.0	0.0
